# Epidemiology and Economic Burden of an Outbreak of Cyclopeptide-Containing Mushroom Poisoning in the West of Iran

**DOI:** 10.3389/fpubh.2022.910024

**Published:** 2022-07-15

**Authors:** Behzad Karami Matin, Mohammad Amrollahi-Sharifabadi, Satar Rezaei, Afshar Heidari, Ali Kazemi-Karyani

**Affiliations:** ^1^Research Center for Environmental Determinants of Health, Health Institute, Kermanshah University of Medical Sciences, Kermanshah, Iran; ^2^Department of Basic Sciences, Faculty of Veterinary Medicine, Lorestan University, Khorramabad, Iran

**Keywords:** epidemiology, economic burden, cyclopeptide, mushroom, poisoning

## Abstract

Little evidence is available on the epidemiological and economical dimensions of certain foodborne diseases such as wild mushroom poisoning. This study aimed to investigate the epidemiology and estimate the costs of poisoning with cyclopeptide-containing mushrooms in Kermanshah province in 2018. In this study, poisoning was investigated in different subgroups. The cost of illness method with a bottom-up approach was used to estimate the poisoning costs. Both direct and indirect costs of the poisoning were included in the analysis. The perspective of the study was society. Required data were obtained from the medical records of Imam Khomeini hospital and completed through a line survey with the patients. Two hundred eighty-three patients were poisoned in Kermanshah due to poisoning with cyclopeptide-containing mushrooms. Of 283 patients, 143 (50.53%) were men and 59.01% of patients were rural residents. About 43% of admissions were out-patient cases and ~40% of patients were hospitalized within 1–3 days. Also, eight patients were pronounced dead in the area. The total cost of poisoning with cyclopeptide-containing mushrooms in Kermanshah province was $ 1,259,349.26. Of that, $ 69,281.65 was related to direct medical costs and $ 10,727.23 was direct non-medical costs. The indirect costs of death were estimated to be $ 1,125,829.7. The current study revealed that there is a significant financial burden due to cyclopeptide-containing mushrooms on patients, the health system, and society as a whole. Further studies are recommended to clarify the epidemiological and economic burden of foodborne illnesses related to wild mushroom poisonings. Sharing the outputs with health authorities, and informing the general public are warranted to reduce the burden of such diseases.

## Introduction

Foodborne diseases (FBDs) are a main public health threat estimated to be the consequence of more than 600 million morbidities and 420,000 mortalities in the world annually, accounting for about 33 million Disability Adjusted Life Years (DALYs) (1). The estimated total economic burden of FBDs is US$ 110 billion in the low- and middle-income countries annually; US$ 95.2 billion for productivity lost and US$ 15 billion for medical costs (2). FBDs can be caused by a vast array of agents from bacterial and viral microorganisms to naturally occurring toxins in food commodities (3). Recent studies have shown that poisonous mushrooms are among the top etiologies of FBDs emphasizing an urgent need to delineate the different aspects of such phenomena for public health planning. Mushroom poisoning by overtaking infectious agents has been the leading cause of foodborne outbreaks in China from 2003 to 2017 with 31.8% of the total of 19,517 outbreaks and resulting in 235,754 ill cases, 107,470 hospitalizations, and 1,457 deaths in this land (4). Another study conducted in South Korea regarding the estimation of the prevalence and burden of FBDs related to poisonings revealed that the mushroom poisonings were among the major etiologies (5).

Mushroom poisoning outbreaks occur frequently following the consumption of wild mushrooms that have been grown due to seasonal climate changes (6). Most cases of mortalities are due to the consumption of mushrooms with a high content of naturally occurring toxins named cyclopeptides (7, 8). Also, cyclopeptide-containing mushrooms are the responsible cause of fatality in developed countries such as the United States with sporadic patterns mostly. Investigation through National Poison Data System from 1999 to 2016 shows that fifty-two (2.9 people per year) fatalities occurred in the United States which in the majority of cases (68–89%) resulted from cyclopeptide-containing mushrooms consumed by older adults accidentally (8).

Most known cyclopeptide toxins included amatoxins and phallotoxins. Cyclopeptides are found in some species of mushrooms, namely, *Amanita* [*A*. *phalloides, A*. *virosa*, and *A*. *Verna*); *Galerina* (*G*. *marginata* and *G*. *venenata*); and *Conocybe* (*C*. *filaris*; (synonym *Pholiotinarugosa*) and *Lepiota* (*L*. *brunneoincarnata*; *L*. *brunneolilacina, L*. *helveola*, and *L. josserandii*)] (9, 10).

The cyclopeptide-containing mushrooms are often mistakenly eaten instead of edible mushrooms. Since the toxins are heat stable, consuming in either raw or cooked way cannot abrogate the nature of toxicity (11). Importantly, these chemicals can induce a three-phased toxidrome that can be life-threatening if timely diagnosis and quick treatment are not provided. The first stage in which the dose does not appear soon after ingestion of the poisoned meal (6–24 h) is the gastrointestinal (GI) phase which may be considered similar to common poisonings. The second phase emerges when the patient recovered apparently through supportive treatments for GI disturbances but hepatic damage is underway. This stage may linger 2–3 days characterized by changes in serum biomarkers of liver damage, coagulopathy, and finally hepatic encephalopathy. The third phase is associated with organ failure and the possibility of death (11).

One might speculate that preventing the consumption of wild mushrooms is the best strategy to avoid morbidity and mortality of poisonous ones. However, the behavior of harvesting and consuming the wild mushrooms may be normal in some parts of the world to the extent that they may be categorized as mycophilic (e.g., Southeast Asia, the Venezuelan Amazon, Slavic countries, and Italy) or mycophobic (e.g., the United Kingdom). People of mycophilic regions are extremely eager in consuming wild mushrooms that originated in their long history and culture while the mycophobic inhabitants scarcely harvested and consumed wild mushrooms (12). Unfortunately, there is no simple method to differentiate between edible and poisonous mushrooms and only experts such as mycologists and toxicologists can discern the *poison* and *remedy*.

In the spring of 2018, an unprecedented foodborne outbreak of mushroom poisoning occurred in Iran, with more than 800 poisoned cases of which more than 300 cases occurred in Kermanshah province, in the west of Iran. This epidemic phenomenon was the result of climate changes and exceptional precipitations accelerating the growth of cyclopeptide-containing mushrooms in Kermanshah (dominantly; *Lepiota brunneioncarnata* was determined by the Department of Botany, Iranian Research Institute of Plant Protection, Agricultural Research, Education and Extension Organization, Tehran, Iran). Nevertheless, the epidemiological and economical dimensions of these cases have not been addressed in the past. Therefore, this study aimed to determine the epidemiology and estimate the economic burden of these poisonings. The findings of the present study will help health policymakers to establish a better preventive program and take more robust measures.

## Methods

This study was conducted by using a descriptive retrospective methodology. Part of the data such as direct costs of treatment for in-patients and out-patients were obtained by studying all 283 registered medical records for poisoning with mushrooms in 2018. Medical record data were obtained from the medical informatics center of Imam Khomeini in Kermanshah province in the west of Iran. This hospital has specialized and subspecialized departments such as eye, ENT, internal medicine, intensive care, emergency poisoning and internal medicine, infectious, burns, dialysis, and specialized and subspecialized clinics to serve the people of Kermanshah and other neighboring provinces in the west of Iran. This hospital has 220 active beds and is a public and referral hospital for poisoning.

Required data were collected using a checklist. Another part of the data was about the indirect costs that were obtained through a line survey. For this purpose, randomly, we called 185 patients or caregivers. If the participants were not available after three calls or refused to be interviewed, the next patient number would be replaced. To estimate the economic burden of poisoning associated with the consumption of cyclopeptide-containing mushrooms, the cost of illness (COI) method based on the human capital method was used (13).

Costs were calculated using a bottom-up and prevalence approach. It should be noted that in cases such as food poisoning due to the short period of the disease, incidence, and prevalence are almost the same. The bottom-up approach usually starts from a specific population group with the disease in question and records all costs associated with that disease. Then, the costs of this subgroup are used to estimate the total costs of the disease in the population. In this study, we calculated the total cost of the disease from the perspective of society. The total economic burden of illnesses consists of two parts, one being the direct costs and the other the indirect costs (14):

(A) Direct costs: These included both medical and non-medical costs. Direct medical costs measure the economic resources used to treat poisoning, namely, out-patient, in-patient, medicine, and other medical costs. Direct non-medical costs included transportation costs, travel costs to medical centers, and accommodation costs in other cities for treatment.

(B) Indirect costs: Human capital approach (HCA) was used to calculate the indirect economic burden of poisoning. In HCA, the value of human capital depends on the future income of patients. In this method, the period that the patient loses due to absence from work is used as a basis for the calculation of indirect cost. Therefore, the higher the income and the longer the absence from work, the higher the indirect cost of the disease. In this study, indirect costs were divided into indirect costs caused by lost days (due to disability and receiving treatment) for patients and caregivers and GDP lost due to premature death. Indirect costs for each family depended on daily income and sick leave, average daily income for each caregiver, and the length of time absent from work for nursing and patient care. The gross domestic product was used to calculate the potential lost production due to premature death. GDP per capita in Iran was obtained from World Bank data (15).

1. Production lost due to premature death: for measuring the production lost the following formula was used:

$$\begin{array}{l} Production\;lost\;due\;to\;premature\;death=\overset{N}{\underset{i=1}{\sum }}\frac{{W\left(1+g\right)}^{i}}{{\left(1+r\right)}^{i}} \end{array}$$

Where *W* is GDP per capita, *g* is the average rate of economic growth in Iran (3.7%), *i* is a year lost due to premature death, and *r* is the discount rate (5%). GDP per capita was considered 6,000 $ according to Iran's GDP in 2017.

2. Production lost due to disability or seeking treatment: This cost is calculated by multiplying the number of hospitalization days or days lost (due to disability or/and seeking treatment) for patients and caregivers by the average daily wage for them.
3. Other indirect costs: Includes some costs such as burial expenses that were calculated for the deceased. Finally, by summing up all types of production lost costs, the total production lost due to poisoning with mushrooms was calculated. For all types of direct and indirect costs, average costs were calculated by different subgroups of patients. Each US dollar is equal to 32,000 Iranian Rials in this study.

## Results

According to data extracted from hospital records, 283 patients were referred to Imam Khomeini hospital in Kermanshah due to poisoning with cyclopeptide-containing mushrooms. Of these, 143 (50.53%) were men. The highest incidence of poisoning was in the age group of 20–39 years, and the lowest was in persons over 60 years old. Out of the total referrals, 43.5% were out-patient and ~40% of patients were hospitalized within 1–3 days. A total of 59.0% of patients were rural residents. Eight patients died due to poisoning. A total of 52.43% of the 185 patients who were interviewed by telephone were men. The characteristics of patients are shown in Table 1.

**Table 1:** Characteristics of people who poisoned by cyclopeptide-containing mushrooms in Kermanshah province, Iran, 2018.

**Variable**		**Medical records' data**		**Survey data**	
		***n***	**%**	***n***	**%**
Gender	Male	143	50.53	97	52.43
	Female	140	49.47	88	47.57
Age group (years)	13–19	28	9.89	17	9.19
	20–39	133	47	89	48.11
	40–59	98	34.63	55	29.73
	≥60	24	8.48	24	12.97
Length of stay (days)	0 (outpatient)	123	43.46	39	21.08
	1–3	113	39.93	103	55.68
	4–6	32	11.31	29	15.68
	7–14	15	5.3	14	7.57
Place of residency	Rural	167	59.01	65	35.14
	Urban	116	40.99	120	64.48
Outcome	Health	275	97.17	177	95.68
	Death	8	2.83	8	4.32

According to hospital records, $ 67,385.10 was spent in the hospital for out-patient and in-patient treatment, of which $ 6,573.52 was franchised. The average cost per patient was $ 223.13 shown in Table 2.

**Table 2:** Direct medical costs of the poisoning treatment in Kermanshah province, Iran (obtained from medical records $).

**Category**	**Direct medical costs**	**Per capita costs**
Total	67385.10	223.13
Co-insurance	6573.52	21.77
Insurance commitments	56038.72	185.56

Per capita, direct medical costs were US$ 236.18 for men and US$ 253.63 for women. The direct non-medical costs for men and women were US$ 33.40 and US$ 42.87, respectively. By age, the highest per capita, direct medical costs (US$ 677.99) and direct non-medical costs (US$ 56.97) were in the age group of 60 years and older. Per capita, direct medical and non-medical costs were positively correlated with a prolonged hospital stay, with per capita, direct medical costs being only US$ 53.93 for out-patient cases, compared with US$ 744.49 for patients hospitalized for 7–14 days. Per capita, direct medical costs were $ 189.34 for the rural residents and US$ 324.67 for the urban population. Per capita, non-medical direct costs were higher for rural than urban (US$ 71.85 vs. US$ 19.52). Direct medical and non-medical costs for the deceased were significantly different from other patients. Per capita, direct costs were $ 268.80.

Regarding the indirect costs associated with disability, women (US$ 91.12) 13- to 19-year-old patients (US$ 115.20), patients with hospitalization duration of 7–14 days (US$ 355.65), and rural patients (US$ 92.77) had higher costs than their counterparts. Per capita, the indirect cost of disability for patients and caregivers was US$ 85.95 and US$ 26.94, respectively. Other indirect costs (average burial costs) were US$ 1,757. Per capita, direct medical and non-medical costs and indirect costs are shown in Table 3.

**Table 3:** Per capita, direct, and indirect costs of poisoning with mushrooms by different characteristics of the patients in Kermanshah province, 2018.

**Variable**		**Direct costs (per capita)**			**Indirect costs (per capita)**				**Total costs (per capita)**
		**Medical**	**Non-medical**	**Total**	**Due to disability**		**Others**	**Total**	
		**(*N* = 283)**	**(*N* = 185)**		**(*****N*** **=** **185)**		**(*N* = 185)**	**(*N* = 185)**	
					**Patients**	**Caregivers**			
Gender	Male	236.18	33.40	269.58	81.27	27.26	72.49	181.02	450.60
	Female	253.63	42.87	296.50	91.12	26.59	79.90	197.61	494.10
Age group (years)	13–19	219.60	39.89	259.49	115.20	39.92	0.00	155.11	414.60
	20–39	162.89	33.46	196.35	75.61	20.38	43.89	139.88	336.23
	40–59	257.11	36.16	293.27	92.59	30.21	139.20	262.00	555.28
	≥60	677.99	56.97	734.96	88.77	34.61	104.17	227.55	962.51
Length of stay (days)	0 (outpatient)	53.93	4.31	58.24	0.53	0.00	0.00	0.53	58.78
	1–3	270.81	39.08	309.89	58.11	19.20	81.92	159.22	469.11
	4–6	652.46	25.26	677.72	171.50	52.85	86.21	310.56	988.28
	7–14	744.49	149.06	893.56	355.65	105.30	223.21	684.17	1577.73
Place of residents	Rural	189.34	71.85	261.19	92.77	30.60	170.67	294.05	555.24
	Urban	324.67	19.52	344.19	82.28	24.96	24.74	131.98	476.17
Result of disease	Health	231.21	14.55	245.75	86.55	26.18	0.00	112.72	358.48
	Death	712.47	554.69	1267.16	72.92	43.88	1757.81	1874.60	3141.76
Total	230.94	37.91	268.84	85.95	26.94	76.01351	188.91	457.75

Four men and four women died due to poisoning. Production lost due to premature deaths was US$ 1,125,829.70. The highest cost of death was in the age group of 59–40 years shown in Table 4.

**Table 4:** Production lost due to premature death from poisoning with mushrooms in Kermanshah province, 2018.

**Age group**	**Number of death**		**Production lost**
	**Male**	**Female**	
13–19	0.00	0.00	0.00
20–39	1.00	1.00	415503.07
40–59	2.00	2.00	558118.46
≥60	1.00	1.00	152208.16
Total	4.00	4.00	1125829.70

**Figure 1:**
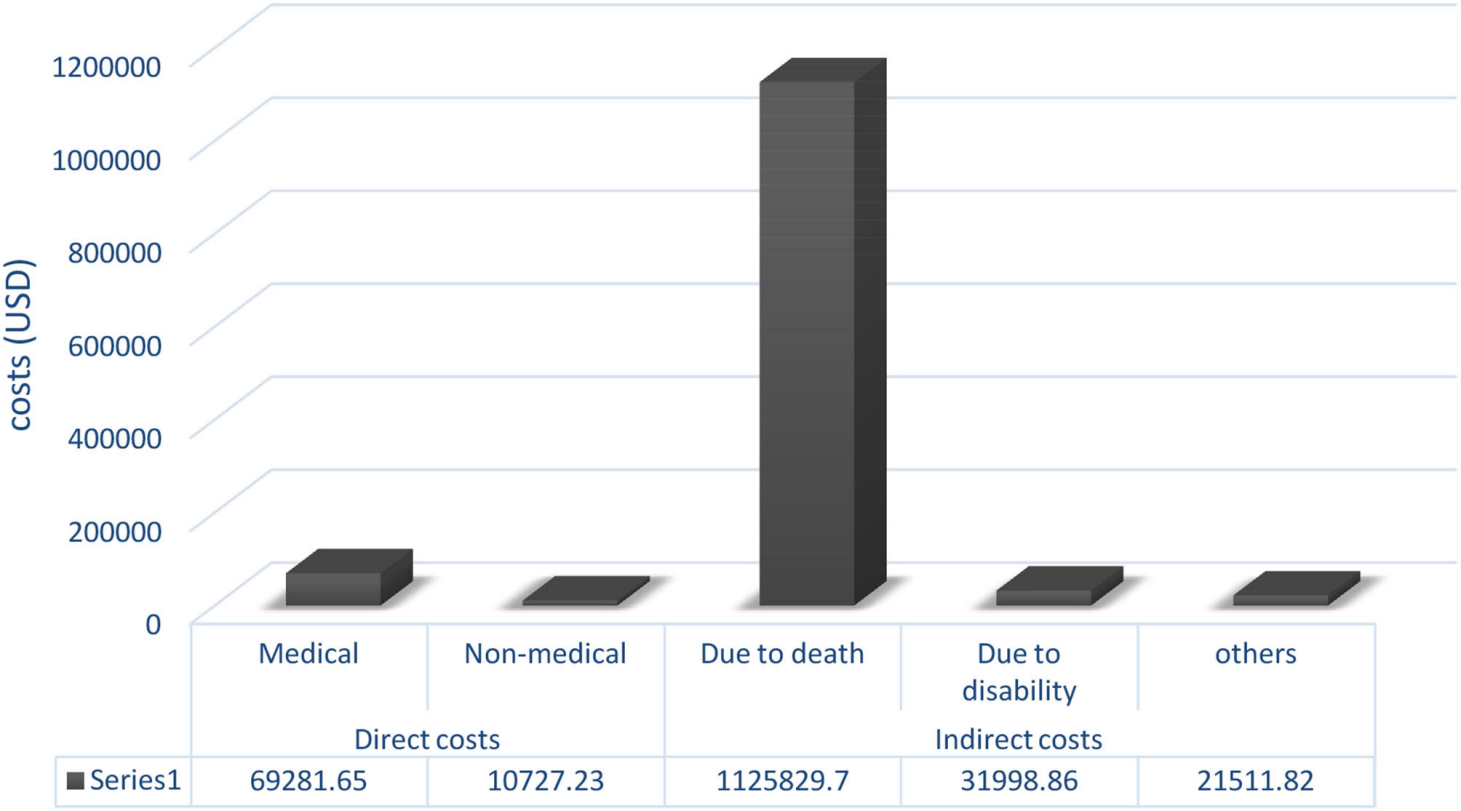
Total costs of poisoning with mushrooms in Kermanshah province, Iran, 2018.

The total cost of poisoning with cyclopeptide-containing mushrooms in Kermanshah province was US$ 1,259,349.26. Of that, US$ 69,281.65 was related to direct medical costs (in-patient and out-patient costs in hospital + other medical costs such as pharmaceuticals), and US$ 10,727.23 was direct non-medical costs. The indirect costs of death were estimated to be US$ 1,125,829.7. The total direct and indirect costs are shown in Figure 1.

Altogether, premature death imposed 90% of the costs of mushroom poisoning, followed by direct medical costs (5% of total costs).

## Discussion

Food poisoning through the consumption of wild mushrooms is considered a public health concern worldwide. However, epidemiological data related to poisonous wild mushrooms vary depending on social factors such as food culture, eating habits, and tendency to be mycophilic, and environmental conditions such as favorable weather and rainfall in different parts of the world. Countries, namely, South Korea, China, Thailand, Russia, Switzerland, Turkey, Nepal, and Italy reported different morbidity and mortality associated with the use of poisonous mushrooms (5, 16–22).

The pattern of wild mushroom poisonings in Iran, as an Asian country, reported different rates of morbidity and mortality in different provinces in previous studies. Pajoumand et al. by reviewing 72,421 poisoned cases of all causes admitted in Loghman-Hakim Hospital Poison Center from 1992 to 2002 found that 0.05% (37 patients) of the population study was due to wild mushroom poisoning (23).

Another study conducted in Mashhad, Iran, from February 2005 to 2011 shows that mushroom poisoning caused a high mortality rate (22%), despite low frequency (prevalence = 0.1%) (24). A recent study by evaluating retrospectively four-year admissions of Razi Hospital, Qaemshahr, Mazandaran, Iran from 2015 to 2018 concluded that a total of 65 patients of wild mushroom poisoning in this region. Also, they found that the major of poisoning cases occurred in the spring season (25).

Our study indicated that of the total of 283 poisoned patients admitted to Kermanshah referral hospital due to poisoning with cyclopeptide-containing mushrooms in spring 2018. Of them, 143 (50.53%) were men and 59.01% of patients came from rural areas. About 43% of admissions were out-patient patients and ~40% of cases were hospitalized within 1–3 days. Also, an amount of 2.83% of the population study (*n* = 8) died due to severe consequences of the disease.

A study by Liu et al. showed that most cases of fatality attributed to the poisonous mushrooms (440 deaths, 54.7%) and occurred mostly in remote, less developed areas in which people believed that consuming wild mushrooms picked from nature is good for their health (26). Research in South Korea during 2008–2012 revealed that mushroom poisoning was at the top list of etiologies of foodborne illnesses. The study showed that the highest occurrence of food poisoning cases belonged to the adults over 40 age (55–59 years old) accounted for 70.6% of all cases (5). Moreover, the study demonstrated the impact of seasonal climate changes on mushroom poisoning.

Altuntas et al. in their study of the records of patients who were admitted to an emergency department of a University hospital in Turkey between January 2008 and December 2012 reported a total of 420 poisoned cases with wild mushrooms. The ratio of men to women was 1/1.5. The patients had a mean age of 46 years. Wild mushroom poisoning of composed of 13.3% of other posing cases. Of the patients, 47.6% were rural residents whereas 38.6% were urban and 13.8% live in city centers. The majority of admissions occurred in autumn (57.6%). Eighty-six percent of poisoning cases were due to the consumption of wild mushrooms picked from nature. Even though most of the patients show mild gastrointestinal symptoms and were discharged after supportive therapy, two cases died and ten cases were referred for liver transplantation. They concluded that wild mushroom poisoning can bring about the critical clinical outcome and thus the public needs to be educated regarding the potential adverse consequences of taking wild mushrooms that are often paper in nature (27). Another study in Turkey that evaluated retrospectively the patients with wild mushroom poisoning between January 2002 and December 2007 in a hospital showed the prevalence of the disease was 9.3%, and the average hospitalization was 3 days (range from 1 to 12 days) (28).

During 2 weeks in December 2016, an outbreak of mushroom poisoning due to the consumption of wild mushrooms was reported in five northern counties of California. A total of 14 patients had been poisoned from taking mushrooms they picked from nature. In comparison with previous annual incidences in the area, the mushroom poisoning that occurred at this time had a much higher incidence and was exceptional. In fact, due to heavy precipitation that happened in the region including San Francisco Bay Area, unusual heavy growth of wild mushrooms (*A*. *phalloides* and related species) had occurred. In sum, 14 poisoned patients reported being admitted for medical treatments. Even though 11 patients recovered after receiving medical treatment, three patients needed liver transplantation as a result of fulminant hepatic failure and one patient succumbed to permanent neurological damage despite receiving liver transplantation. Therefore, the average length of stay in the hospital was as high as almost 9.36 days indicating that this wild mushroom poisoning imposed a relatively long stay in the hospital (on average; 6 days of hospitalization for 11 patients vs. ~21.7 days for 3 patients who received liver transplantation) (29).

To our knowledge, this is the first COI study on the economic impact of wild mushroom poisonings in our country and also worldwide. We adopted the COI method to estimate the economic burden of the mentioned disease because it is one of the most popularly accepted methods employed to estimate the total economic costs of a disease (30). We estimated both direct costs (medical and non-medical) and indirect costs to provide a perspective for society. However, several reports in the literature limited their economical studies of poisonings on either medical costs in specific wards of hospitals [emergency department (31) and intensive care unit (32–34)]. In this respect, it is implied that our study has provided a broader scope.

Sut et al. estimated the average ICU costs for acute poisonings per day as $344 while the cost per ICU stay was valued as high as $821 that they concluded that was lower in comparison with the US costs (the US $925) in Turkey during 2002–2006. Their study shows that admitting acute poisoning cases imposed a substantial cost on the healthcare system in particular ICU wards in Turkey (35).

In our study, the total costs of the mushroom poisoning outbreak were estimated to amount of $ 1,259,349.26. We found that the amount of $ 69,281.65 was allocated to direct medical costs. The amount of $ 10,727.23 was related to direct non-medical costs. The indirect costs of death were estimated to be $ 1,125,829.7.

Descamps et al. calculated the direct costs of poisoned cases admitted to a referral university hospital in Ghent, Belgium, for both the government and the patients in 2017. They found that a total of 1,214 cases were admitted due to acute poisoning, of which 96.9% (1,175) were under the cover of insurance (mandatory health and disability insurance in Belgium). Of all admitted patients, 54.2% received out-patient care, 24.7% were forced to stay for 24 h in the emergency department for close observations, 17.8% were hospitalized, and 3.3% were referred to the intensive care unit. The total direct cost for the treatment of all cases of poisoning (1,175 poisoned patients) was $1,830,870, of which 13.1% were allocated to the emergency department costs and 86.9% went to hospitalization costs. They show that the cost of poisoning is considerable for the government and patients and that the length of hospital stay and accommodation caused a significant share of all costs. However, they did not include the indirect costs of poisoned patients and thus they may underestimate the economic burden of poisoning on their society (36).

Krajewski et al. by investigating an analysis of poisoned cases admitted in Illinois hospitals in 2010, found that there was a total of 425,491 poisoned cases that 222,339 of them were in-patients (52.3%) with an average length of stay of 5.5 days in the hospital. The average total hospital costs was $ 3,398 ± 4,081 in out-patients (median ± $ 2,152) and $ 32,391 ± 60,115 for in-patients (median ± $ 16,396). The total hospital cost for all out-patient cases was $ 690,400,129 and $ 7,201,628,216 for in-patient patients which translated to an estimate of about $7.9 billion in the total cost (37).

Our study shows that the two major shares of costs of mushroom poisoning outbreaks are attributed to the premature deaths resulting in the loss of productivity, and then medical costs. Premature death imposed 90% of the costs of mushroom poisoning, followed by direct medical costs (5% of total costs). These findings are in agreement with previous economic studies on foodborne outbreaks (38–40). Of note, economic models have predicted that the costs of foodborne outbreaks can be varied in terms of different etiologies. Agents that cause an increase in the length of stay in hospital and mortality and thus higher loss of productivity can be imposed in higher economic burden to the society in comparison with agents that accrued in a lower loss of productivity due to shorter stay in hospital and no deaths (41).

## Conclusion

Our study shows that there is a significant financial burden due to cyclopeptide-containing mushrooms on patients, the health system, and society as a whole. The present study results will be a useful tool for health policymakers to establish suitable frameworks for the prevention and control of seasonal food poisonings related to the ingestion of cyclopeptide-containing mushrooms in the future. Further studies are needed to elucidate the epidemiological and economic burden of foodborne illnesses related to wild mushroom poisonings. Providing gratuitous consultation from mycology and toxicology expects for educating people to be able to differentiate between poisonous and edible mushrooms before harvesting and consumption, can reduce the burden of wild mushroom poisonings.

## Data Availability Statement

The raw data supporting the conclusions of this article will be made available by the corresponding authors with reasonable requests.

## Ethics Statement

The studies involving human participants were reviewed and approved by the Ethics Committee of Kermanshah University of Medical Sciences (IR.KUMS.REC.1397.427). The patients/participants provided their written informed consent to participate in this study.

## Author Contributions

BK: conceptualization, methodology, funding acquisition, and writing—review and editing. MA-S: investigation, methodology, and writing—review and editing. SR: formal analysis, software, and validation. AH: visualization and data curation. AK-K: supervision, project administration, software, and funding acquisition. All authors contributed to the article and approved the submitted version.

## Funding

This study was extracted from the Kermanshah University of Medical Sciences approved project and was funded and supported by the Research Deputy of Kermanshah University of Medical Sciences (Grant No. 97459).

## Conflict of Interest

The authors declare that the research was conducted in the absence of any commercial or financial relationships that could be construed as a potential conflict of interest.

## Publisher's Note

All claims expressed in this article are solely those of the authors and do not necessarily represent those of their affiliated organizations, or those of the publisher, the editors and the reviewers. Any product that may be evaluated in this article, or claim that may be made by its manufacturer, is not guaranteed or endorsed by the publisher.

## Abbreviations

COI: Cost of illness
GDP: Gross domestic product
US $: United States dollar.
